# Impact of Mycotoxins Secreted by *Aspergillus* Molds on the Inflammatory Response of Human Corneal Epithelial Cells

**DOI:** 10.3390/toxins9070197

**Published:** 2017-06-22

**Authors:** Yélian Marc Bossou, Youssra Serssar, Amel Allou, Sandrine Vitry, Isabelle Momas, Nathalie Seta, Jean Menotti, Sophie Achard

**Affiliations:** 1 Environmental Epidemiology Unit, Paris-Descartes University, Sorbonne Paris Cité, EA 4064, 75006 Paris, France; bossoumarc1@gmail.com (Y.M.B.); serssaryoussra2gmail.com (Y.S.); amel_allou@yahoo.fr (A.A.); isabelle.momas@parisdescartes.fr (I.M.); nathalie.seta@parisdescartes.fr (N.S.); sophie.achard@parisdescartes.fr (S.A.); 2 Viral Neuroimmunology Unit, Pasteur Institute, 75015 Paris, France; Sandrine.vitry@pasteur.fr; 3 Laboratory of Biochemistry, Bichat University Hospital, AP-HP, 75018 Paris, France; 4 Laboratory of Parasitology-Mycology, Saint-Louis University Hospital, AP-HP and Paris-Diderot University, Sorbonne Paris Cité, 75010 Paris, France

**Keywords:** mycotoxin, aflatoxin B_1_, gliotoxin, in vitro, ocular surface, inflammatory response, cellular impedance

## Abstract

Exposure to molds and mycotoxins not only contributes to the onset of respiratory disease, it also affects the ocular surface. Very few published studies concern the evaluation of the effect of mycotoxin exposure on ocular cells. The present study investigates the effects of aflatoxin B_1_ (AFB_1_) and gliotoxin, two mycotoxins secreted by *Aspergillus* molds, on the biological activity of the human corneal epithelial (HCE) cells. After 24, 48, and 72 h of exposure, cellular viability and inflammatory response were assessed. Both endpoint cell viability colorimetric assays and continuous cell impedance measurements, providing noninvasive real-time assessment of the effect on cells, were performed. Cytokine gene expression and interleukin-8 release were quantified. Gliotoxin appeared more cytotoxic than AFB_1_ but, at the same time, led to a lower increase of the inflammatory response reflecting its immunosuppressive properties. Real-time cell impedance measurement showed a distinct profile of cytotoxicity for both mycotoxins. HCE cells appeared to be a well-suited in vitro model to study ocular surface reactivity following biological contaminant exposure. Low, but persistent inflammation, caused by environmental factors, such as fungal toxins, leads to irritation and sensitization, and could be responsible for allergic manifestations which, in turn, could lead to mucosal hyper-reactivity.

## 1. Introduction

Mycotoxins are common contaminants of agricultural crops produced by several genera of fungi, such as *Aspergillus* molds, in response to both intrinsic and extrinsic factors, such as, respectively, toxigenic status of fungi and temperature and humidity [1]. These toxins can enter into the food chain, leading to adverse effects on animal and human health at low concentrations [2]. The United Nations-affiliated Food and Agriculture Organization has assessed that an average of 25% of global agricultural commodities may be contaminated with mycotoxins [3].

Fungi and their mycotoxins are ubiquitous in the environment and, once produced, these contaminants are adsorbed onto airborne dusts, leading to major public health issues. Mycotoxin toxicity via the ingestion route has been extensively studied [4,5,6,7], such as aflatoxins that play an important role in the development of hepatocellular carcinoma [5,8]. The respiratory route has been recognized in the past two decades as an important route of exposure, especially for workers in corn storage facilities and in animal farms [9,10,11]. Indeed, some studies have established an association between low-level exposure to molds and mycotoxins, and asthma or chronic airway inflammation, especially among workers in an agricultural setting [9,12]. Such exposure is related to the onset of farmers’ lung disease [13], hypersensitivity pneumonia, and allergic bronchopulmonary aspergillosis [14]. Molds belonging to the *Aspergillus* genus and producing mycotoxins, such as aflatoxins or gliotoxin, contribute to the onset of respiratory diseases by the exposure of nasal, bronchial, and alveolar epithelia. This type of exposure also concerns the ocular surface, leading to irritations or allergic manifestations [15].

In common with epidemiological and clinical studies, toxicological studies are traditionally based on animal tests. However the 3R principles that promote alternatives to animal experimentation are now particularly encouraged and in vitro studies using cell culture are usually implemented in toxicology [16]. Most in vitro studies aiming at assessing the impact of mycotoxins have used alveolar, bronchial, or nasal epithelial cells [17,18], whereas only very few studies have used ocular epithelial cells to explore house dust-induced toxicity [19,20] and, to our knowledge, no study has explored mycotoxin-induced toxicity on ocular epithelial cells.

To test the impact of the exposure of the ocular surface to mycotoxins, we assessed the effects of two mycotoxins produced by *Aspergillus* molds, aflatoxin B_1_ (AFB_1_), and gliotoxin on human corneal epithelial (HCE) cells.

## 2. Results

In order to evaluate the effects of AFB_1_ and gliotoxin on the ocular cells (HCE), we conducted two experimental approaches. In a first approach, using classical in vitro assays, both cellular viability and inflammatory response, interleukin-8 (IL-8) release, and gene expression quantification of seven inflammatory markers were assessed at different times and concentrations of mycotoxins. In a second approach, real-time monitoring of cellular impedance reflecting the kinetics of toxicity was implemented using xCelligence technology.

### 2.1. Cellular Viability and Inflammatory Response of HCE Cells after AFB_1_ and Gliotoxin Exposures

Seventy-two hours after seeding, HCE cells were exposed to various concentrations of AFB_1_ (from 0.5 to 128 µg/mL) and gliotoxin (from 2 to 500 ng/mL) for 24, 48, or 72 h. After these exposure times, a 3-(4,5-dimethyl-2-thiazolyl)-2,5-diphenyl-2*H*-tetrazolium bromide (MTT) test was performed on cell monolayer in order to assess cellular viability, and IL-8 secretion was quantified in culture medium to evaluate the inflammatory response. The two controls, solvent-control (DMSO) and incubator-control, were performed under the same experimental conditions.

**Figure 1:**
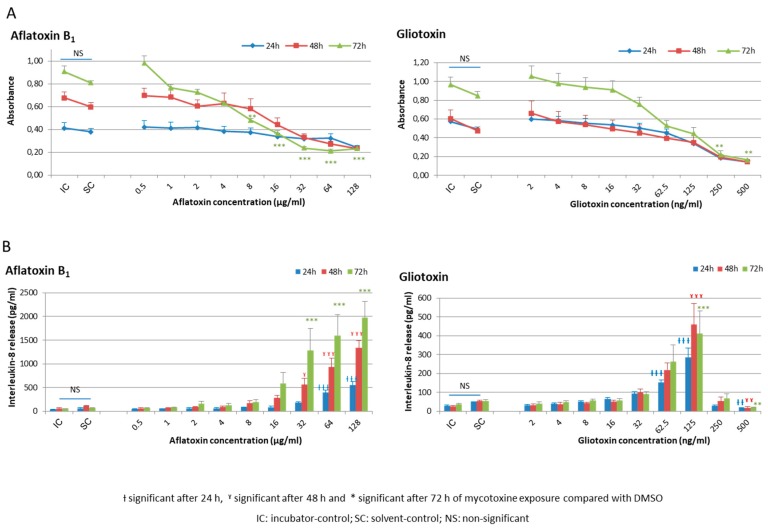
Cellular viability and inflammatory response of HCE cells after mycotoxin exposure. (**A**) Cellular viability after aflatoxin B_1_ and gliotoxin exposure using MTT assay. After 24, 48, or 72 h of exposure to nine different concentrations of aflatoxin B_1_ or gliotoxin, to DMSO alone (solvent-control for aflatoxin B_1_ and for gliotoxin, at concentrations of 1.28% and 0.01%, respectively) or to medium alone (incubator-control), the culture medium was harvested and MTT was deposited in each well. After 3 h of incubation at 37 °C and solubilization of formazan salts with DMSO, the absorbance was measured at 490 nm; and (**B**) interleukine-8 release after aflatoxin B_1_ and gliotoxin exposure using an ELISA assay. The quantification of IL-8 release was performed in the harvested culture media with an ELISA assay kit.

No effect on both cellular viability and inflammatory response was observed after DMSO exposure compared with the incubator-control (Figure 1 and Figure S1).

Concerning AFB_1_, no decrease of cellular viability was observed after 24 h of exposure whatever the concentration tested. After 72 h of exposure, a significant reduction in cell viability compared with solvent-control was seen for the 8 µg/mL concentration and beyond (Figure 1A).

Concerning gliotoxin, a progressive decrease in cellular viability was observed, which became significant after 24 h and 72 h exposure at 250 and 500 ng/mL, respectively (Figure 1A).

For more clarity, cellular viability results expressed as absorbances in Figure 1A to better represent the growth kinetics between 24, 48, and 72 h of culture and for a better statistical approach are also expressed as the percentage of viability compared to the solvent-control in Figure S2.

After 48 and 72 h of exposure, a significant increase in IL-8 release was observed at concentrations of 32 µg/mL and 62.5 ng/mL, respectively, for AFB_1_ and gliotoxin (Figure 1B). The high toxicity noted with the MTT test after gliotoxin exposure at 250 and 500 ng/mL was confirmed with a dramatic drop of cytokine release (Figure 1B).

The gene expression of seven pro-inflammatory markers produced by HCE cells was quantified after 48 h of exposure to AFB_1_ and gliotoxin, respectively, at 16 µg/mL and 125 ng/mL. These two concentrations were chosen because they were associated with a significant increase of IL-8 secretion with a moderate decrease in cellular viability. Solvent-control and incubator-control were conducted under the same experimental conditions. DMSO exposure did not affect gene expression when compared to incubator-control (data not shown).

**Figure 2:**
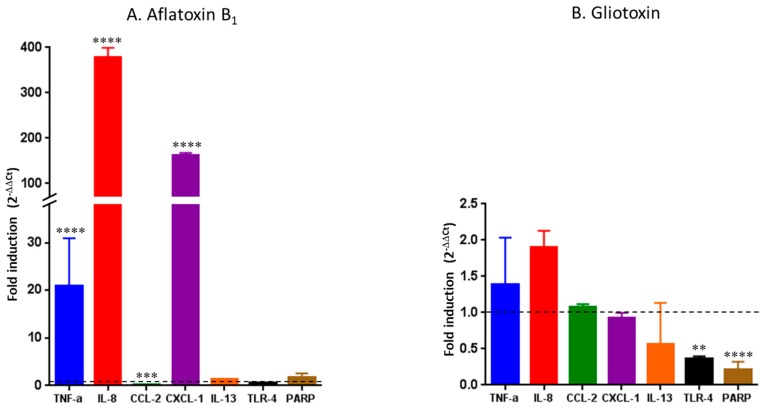
Gene expression of seven proinflammatory markers after a 48 h exposure of HCE cells to 16 µg/mL of aflatoxin B_1_ (**A**) or 125 ng/mL of gliotoxin (**B**). Results are expressed as fold induction versus incubator-control. **** *p* < 0.0001; *** *p* = 0.0002; ** *p* = 0.0041.

Exposure to AFB_1_ at 16 µg/mL induced a significant and marked increase in IL-8, C-X-C motif chemokine ligand 1 (CXCL-1), and tumor necrosis factor-α (TNF-α) gene expression (*p* < 0.0001) with, respectively, 380-fold, 160-fold, and 21-fold inductions, and a significant 0.26-fold decrease in the C-C motif chemokine ligand 2 (CCL-2) gene expression (*p* = 0.0002). The gene expression of the other cytokines of interest (interleukin-13 (IL-13), Toll-like receptor 4 (TLR-4), and poly (ADP-ribose) polymerase (PARP)) was not affected (Figure 2A).

Exposure to gliotoxin at 125 ng/mL induced a non-significant slight increase in IL-8 and TNF-α gene expression with, respectively, 1.9-fold and 1.4-fold inductions, and a significant decrease in TLR-4 (*p* = 0.0041) and PARP (*p* < 0.0001) gene expression. The gene expression of CCL-2, CXCL-1, and IL-13 was not affected by gliotoxin exposure (Figure 2B).

Globally, IL-8 and TNF-α gene expression was respectively 200-and 15-times higher after AFB_1_ exposure than after gliotoxin exposure (*p* < 0.0001).

### 2.2. Kinetics of Cellular Impedance after Exposure of HCE Cells to AFB_1_ or Gliotoxin

In order to monitor the kinetics of toxicity after exposure to both mycotoxins, a noninvasive label-free real-time monitoring of cellular impedance with interdigitated gold electrode-containing microtiter plates was performed [21,22].

After the adhesion and proliferation phases, HCE cells reached the stationary phase after about 60 h. At this time, HCE cells were exposed to different concentrations of AFB_1_ (8, 16, 32, 64, or 128 µg/mL) or gliotoxin (62.5, 125, 250, or 500 ng/mL). Solvent-control (DMSO) and incubator-control were performed under the same experimental conditions.

**Figure 3:**
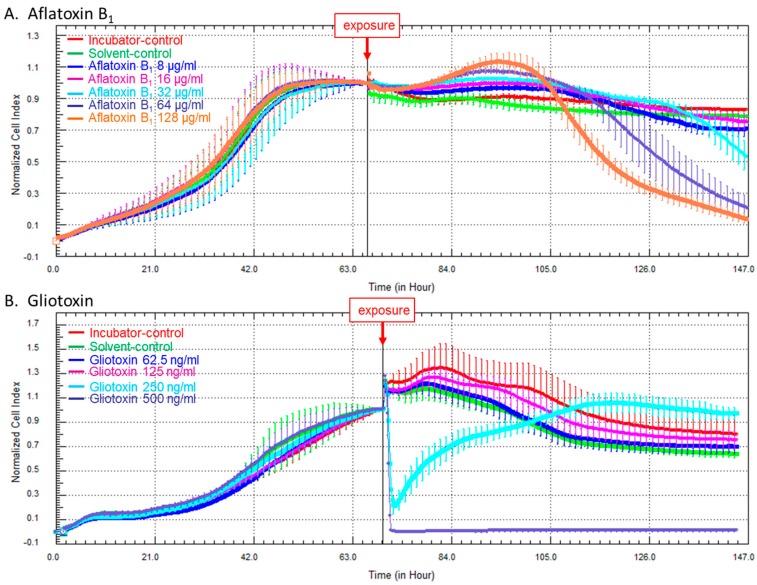
Effect of aflatoxin B_1_ (**A**) and gliotoxin (**B**) on HCE cell survival measured by cell impedance-based technology. (**A**) Cells were exposed to 8, 16, 32, 64, or 128 µg/mL of aflatoxin B_1_ or to the concentration of DMSO alone equivalent to the DMSO concentration in 128 µg/mL of aflatoxin B_1_; and (**B**) cells were exposed to 62.5, 125, 250, or 500 ng/mL of gliotoxin or to the concentration of DMSO alone equivalent to the DMSO concentration in 500 ng/mL of gliotoxin. The error bars on the profiles represent 95% confidence intervals.

No effect of DMSO exposure compared with incubator-control was observed (Figure 3).

Exposure to 8 or 16 µg/mL of AFB_1_ induced no significant effect compared with solvent-control. Exposure to 32, 64, or 128 µg/mL of AFB_1_ led to a progressive, but significant, decrease of cell indices from, respectively, 70 h, 50 h, or 40 h after challenge, without subsequent recovery, reflecting cell death (Figure 3A).

Exposure to 62.5 or 125 ng/mL of gliotoxin had no significant effect compared with solvent-control. Exposure to 250 ng/mL of gliotoxin induced a significant decrease of cell indices, followed by subsequent cell recovery, whereas exposure to 500 ng/mL of gliotoxin led to a sharp and definitive decrease of cell indices, indicating a fatal issue for the HCE cells (Figure 3B).

## 3. Discussion

Using an original in vitro approach combining both end-point tests and real-time monitoring of cellular impedance, we have evaluated the impact of two mycotoxins on the cellular viability and the inflammatory response of the ocular epithelium represented by the HCE cells. The anatomical position of the cornea means that it is directly in contact with environmental pollutants. Consequently, HCE cells are well adapted to the assessment of the biological effects of numerous airborne compounds on the ocular surface [23]. Our results have highlighted a concentration- and time-dependent modulation of the inflammatory response, with an increase in both gene expression of inflammatory markers (IL-8, CXCL-1, and TNF-α), and IL-8 release after AFB_1_ or gliotoxin exposure. Furthermore, a loss of HCE cell viability has been shown after high exposure levels of AFB_1_ or gliotoxin. Real-time cell impedance measurement to further evaluate the kinetics of cellular toxicity of the two mycotoxins was also performed, and confirmed the higher toxicity of gliotoxin compared with AFB_1_. This approach was very interesting because it allowed the estimation of the cellular toxicity with a noninvasive real-time assessment of the xenobiotic effect. Such information cannot be obtained by classical in vitro toxicity tests, such as tests based on the reduction of tetrazolium salt (MTT assay), which only give an end-point assessment. This cell impedance measurement-based technology can be used in order to complete the evaluation of the toxicity of various compounds using in vitro study, in accordance with the strategy of replacement of laboratory animals [24].

The concentrations of mycotoxins associated with inflammation but low cytotoxicity were 32 µg/mL and 125 ng/mL for AFB_1_ and gliotoxin, respectively. Although the AFB_1_ cytotoxicity appeared to be lower as compared to gliotoxin, the inflammatory response was more important after AFB_1_ exposure than after gliotoxin exposure. This paradoxical result could be explained by the immunosuppressive properties of gliotoxin. In an in vitro study, Pahl et al. [25] evidenced that gliotoxin inhibited the activation of NF-κB transcription factor, leading to an inhibition of the transcription of pro-inflammatory cytokines and an inhibition of the inflammatory response. Additionally, the reduced IL-8 release at the highest concentrations (250 and 500 ng/mL) is to be related to the reduced cell viability evidenced by the MTT test at these concentrations. The high toxicity we evidenced with gliotoxin is in accordance with previous reports on the toxicity of gliotoxin on other cell lines [26]. This toxicity may act through apoptosis and necrosis [26].

Most of the available data in the literature on the relation between the ocular surface and inflammation concern benzalkonium chloride (BAK), an antimicrobial preservative used in many ophthalmic solutions, known to promote the NF-κB pro-inflammatory pathway [27]. BAK is also used for this anti-inflammatory effect to improve the symptoms of dry eye syndrome [28]. Very few data are available on the impact of environmental pollutants on the ocular surface [29]. To the best of our knowledge, the present in vitro study is the first study to evidence the impact of mycotoxins on the gene expression of several cytokines involved in the inflammation of the ocular surface.

A dose-dependent induction of the gene expression of IL-8, CXCL-1, and TNF-α was observed in our study, more significantly after AFB_1_ exposure than after gliotoxin exposure. These cytokines lead to the activation and migration of leukocytes, mainly neutrophils, and to the production of other pro-inflammatory cytokines, contributing to the implementation of ocular damage. A similar dose-dependent induction of the gene expression of IL-8 and TNF-α was observed for BAK [30]. Such an induction of the gene expression of IL-8 and TNF-α was also evidenced in patients with Sjögren syndrome [31].

The main function of CXCL-1 is to recruit neutrophils during the inflammation process [32]. Our data reveal that CXCL-1 gene expression is 160-fold induced when compared to control following the exposure of HCE cells to 16 µg/mL of AFB_1_. An in vivo study on rats exposed for 16 h to *Aspergillus fumigatus* revealed a 3.5-fold induction of this chemokine when compared to control [33]. However, to our knowledge, no study on CXCL-1 gene expression has been reported on animals exposed to aflatoxigenic fungi. Moreover, in vivo conditions could reduce the duration of contact of the mycotoxin with corneal epithelial cells due to ciliary beat and watering eyes [34], making it difficult to compare the expression levels found in in vivo and in in vitro studies. Nevertheless, we can hypothesize that CXCL-1 might be involved in pro-inflammatory signaling pathway following environmental exposure.

Contrary to this, the gene expression of CCL-2 was not induced by gliotoxin and was even decreased by AFB_1_; as CCL-2 is a chemoattractant chemokine for monocytes [35], we can hypothesize monocytes to be less involved in the inflammation process following exposure to both mycotoxins. The gene expression of IL-13, a cytokine known to contribute to airway allergies [36,37], was not induced either. One would expect an induction of TLR-4 gene expression after direct exposure to fungi as the involvement of this receptor in the immune response to *Aspergillus fumigatus* was evidenced previously [38,39]. However, in our conditions, no induction of TLR-4 gene expression was observed after exposure of HCE cells to AFB_1_ and even a decrease in TLR-4 gene expression was observed after exposure to gliotoxin.

The impact of mycotoxins on the respiratory tract has been studied by several authors, mainly in terms of metabolic activity. Thus, tracheal, laryngeal, nasal, and conjunctival mucosa have been shown to be able to bioactivate AFB_1_ to AFB_1_-8,9-epoxide, which may promote toxicity and carcinogenicity as already found in hepatic tissue [40]. Gliotoxin has been shown to have immunosuppressive properties in vitro and in vivo, to inhibit phagocytosis by macrophages, and to cause apoptosis in primary and secondary lymphoid organs [41], contributing to respiratory illness. In a murine asthma model, respiratory exposure to gliotoxin has been shown to decrease the production of IL-12 by dendritic cells and of IFN-γ by T cells and to increase Th2 cytokine levels, leading to a Th2-driven allergic immune response [18].

Given the in vitro effects of AFB_1_ and gliotoxin on corneal epithelial cells, in order to prevent from toxic effects workers with airborne exposure to mycotoxins, such as workers in corn storage facilities or in animal farms, wearing eye protection might be discussed in addition to wearing a mask.

## 4. Conclusions

Taken as a whole, our results on the inflammatory response of HCE cells after exposure to both mycotoxins could be related to ocular irritation or sensitization, leading to allergic manifestations such as mucosal hyperactivity, which might be due to low, but persistent, inflammation in the presence of environmental factors such as fungal toxins [42]. As the ocular allergy symptoms are almost always combined with rhinitis [43], and as nasal and ocular mucosa belong to a complex mucosal and lymphoid tissue network [44], it would be of interest in future work to study the relation between the ocular surface and nasal mucosa in the development of the inflammatory reaction.

## 5. Experimental Section

### 5.1. Chemicals and Reagents

Dulbecco’s modified Eagle’s medium (DMEM), Ham F-12 medium, fetal calf serum (FCS), glutamine, penicillin-streptomycin, trypsin, and dimethyl sulfoxide (DMSO) were purchased from Thermo Fisher Scientific (Waltham, MA, USA). The DuoSet kits for ELISA assays were produced by R and D Systems (Minneapolis, MN, USA). AFB_1_, gliotoxin and 3-(4,5-dimethyl-2-thiazolyl)-2,5-diphenyl-2*H*-tetrazolium bromide (MTT) were purchased from Sigma-Aldrich (Saint-Quentin Fallavier, France).

### 5.2. Cell Line and Culture Conditions

Human corneal epithelial (HCE) cell line was a kind gift from Dr. F. Brignole (Institut de la Vision, Université Pierre et Marie Curie, Paris, France). HCE cells were cultured in DMEM:Ham F-12 (50:50) supplemented with 5% FCS, 1% glutamine and 1% antibiotics (penicillin 100 IU/mL and streptomycin 100 µg/mL). Cells were seeded at a density of 2 × 10^4^ cells/mL and incubated at 37 °C under 5% CO_2_. Culture medium was changed 24 h after seeding and then every three days. When the confluence had been reached, cells were harvested with trypsin and seeded in a new flask.

### 5.3. Cell Exposure to Mycotoxins

AFB_1_ and gliotoxin were first dissolved in DMSO. Then, these stock solutions of AFB_1_ or gliotoxin were diluted in DMEM:Ham F-12 (50:50) without FCS. Cell exposure occurred 72 h after seeding (of 2 × 10^4^ cells per mL) when the confluence was close to 70–80%. At this time, culture media were removed and replaced with culture media containing mycotoxin at nine different concentrations, from 0.5 to 128 µg/mL AFB_1_ and from 2 to 500 ng/mL gliotoxin. Two controls were considered: solvent-control (DMSO), tested at concentrations equivalent to the DMSO concentrations in the different mycotoxin serial dilutions, and incubator-control without exposure. Table 1 summarizes the various concentrations tested. The concentration ranges were chosen in view of the concentrations found to be toxic or to induce inflammatory reaction in the literature on other cell lines [6,7,18,26,45,46]. Cells were exposed for 24, 48, and 72 h to assess both cellular viability on the cell monolayer and IL-8 release in the culture medium. Each condition was tested in quadruplicate in four independent experiments.

The gene expression of seven pro-inflammatory markers (TNF-α, IL-8, CCL-2, CXCL-1, IL-13, TLR-4, and PARP) produced by HCE cells was quantified on the culture monolayer by RT-qPCR after 48h of exposure to AFB_1_ or gliotoxin, respectively, at 16 µg/mL or 125 ng/mL.

For the real-time measurement of cell proliferation, HCE cells were seeded at a density of 8 × 10^3^ cells per well in wells containing gold electrodes (xCELLigence 16-well E-plates, ACEA Biosciences, San Diego, CA, USA). Then, the E-plates were placed under the incubator at 37 °C and 5% CO_2_. After a proliferation phase, HCE cells were exposed to various concentrations of AFB_1_ (8, 16, 32, 64, or 128 µg/mL) or gliotoxin (62.5, 125, 250, or 500 ng/mL) throughout the time of the experiment. Two controls were considered: solvent-control (DMSO) tested for each mycotoxin and incubator-control without exposure. These assays were performed in duplicate in two separate experiments.

### 5.4. Cell Viability

After each exposure time, the 100-µL culture medium was harvested and 100 µL of MTT at a concentration of 5 mg/mL were deposited in each well on the monolayer culture. After 3 h incubation at 37 °C, formazan salts produced by functional cells were solubilized with DMSO. The absorbance was measured at 490 nm using a multiscan plate reader (Multiskan-EX, Thermo Scientific, Waltham, MA, USA), and was proportional to the quantity of functional cells.

### 5.5. Dosage of IL-8 Release

The quantification of IL-8 release was performed in the harvested culture media with ELISA assays kits (R and D Systems, Lille, France) following the manufacturer’s instructions. Concentrations were expressed in pg/mL. The assay lower limit of detection was 31.25 pg/mL.

### 5.6. Quantification of the Gene Expression of Seven Pro-Inflammatory Cytokines

The gene expression of seven cytokines (tumor necrosis factor α (TNF-α), interleukin-8 (IL-8), chemokine (C-C motif) ligand 2 (CCL-2), chemokine (C-X-C motif) ligand 1 (CXCL-1), IL-13, Toll-like receptor-4 (TLR-4), and poly (ADP-ribose) polymerase (PARP)) was quantified by quantitative real-time reverse transcription PCR (RTqPCR). Briefly, RNA extraction was performed on cell lysates with the RNeasy Plus Mini Kit (Qiagen, Courtaboeuf, France). RNA concentrations were measured with Nanodrop ND-200 (Thermo Fisher Scientific, Courtaboeuf, France) and adjusted to 100 ng/µL. Reverse transcription was carried out with the high capacity cDNA reverse transcription Kit (Thermo Fisher Scientific) with 1 × RT Buffer, 1 × dNTP Mix, 1 × random primers, 1 µL of MultiScribe reverse transcriptase and 1000 ng of RNA in a final volume of 20 µL. The gene expression levels of TNF-α, IL-8, CCL-2, CXCL-1, IL-13, TLR-4, and PARP were determined with the Hs99999043_m1, Hs00174103_m1, Hs00234140_m1, Hs00236937_m1, Hs00174379_m1, Hs00152939_m1, and Hs00365416_m1 inventoried TaqMan gene expression assays (Thermo Fisher Scientific), respectively. The TATA box binding protein (assay ID: Hs00427620_m1) was used as an endogenous control. All assays followed an intron spanning design so as not to show any cross-reactivity with genomic DNA. Real-time PCR assays were carried out in an Applied Biosystems 7500 PCR system (Thermo Fisher Scientific) in a 20-µL final volume containing 1 × TaqMan Gene Expression Master Mix, 1 × TaqMan gene expression assay primer and probe mix (Thermo Fisher Scientific), and 100 ng of cDNA. Thermal cycling conditions were: 50 °C for 2 min and 95 °C for 10 min, followed by 50 cycles of 95 °C for 15 s and 60 °C for 1 min. Results were expressed through the 2^−ΔΔ*C*t^ method [47].

### 5.7. Real-Time Measurement of Cell Impedance

HCE cells were seeded at a density of 8 × 10^3^ cells per well in wells containing gold electrodes (xCELLigence 16-well E-plates, ACEA Biosciences, San Diego, CA, USA). During culture, real-time impedance cell measurement was performed, reflecting cell surface adhesion to gold electrodes. Results were expressed as cell index (CI) values defined as (R_n_ − R_b_)/15 where R_n_ is the cell-electrode impedance of the cell-containing well and R_b_ is the background impedance of the well with the media alone, recorded each 15 min throughout the entire experiment duration. All CIs were normalized to the CI measured just before mycotoxin exposure.

### 5.8. Statistical Analyses

For MTT and ELISA tests, one-way analysis of variance (ANOVA) with Bonferroni’s multiple comparisons test was performed in order to compare the results after exposure to the different concentrations of mycotoxins or DMSO alone. For gene expression studies, two-way ANOVA with Bonferroni’s multiple comparisons test was performed. Both analyses were performed with Prism version 6.04 software (GraphPad Software, La Jolla, CA, USA). A *p* < 0.05 was considered statistically significant.

## Figures and Tables

**Table 1:** Various concentrations of aflatoxin B_1_ (AFB_1_), gliotoxin or DMSO tested.

Aflatoxin B_1_ (µg/mL)	% DMSO in Culture Medium *	Gliotoxin (ng/mL)	% DMSO in Culture Medium *
0.5	0.005	2	4 × 10^−5^
1	0.01	4	8 × 10^−5^
2	0.02	8	1.6 × 10^−4^
4	0.04	16	3.2 × 10^−4^
8	0.08	32	6.4 × 10^−4^
16	0.16	62.5	1.25 × 10^−3^
32	0.32	125	2.5 × 10^−3^
64	0.64	250	5 × 10^−3^
128	1.28	500	0.01

Stock solutions: AFB_1_: 10 mg/mL; gliotoxin: 5 mg/mL. * equivalent concentration.

## Supplementary Materials

The following are available online at www.mdpi.com/2072-6651/9/7/197/s1. Figure S1: Cellular viability after 24 h, 48 h or 72 h of exposure to aflatoxin B1, to gliotoxin or to DMSO alone using MTT assay, Figure S2: Cellular viability of HCE cells using MTT assay after 24 h, 48 h or 72 h of exposure to nine different concentrations of aflatoxin B_1_ or gliotoxin.
