# Supplementary material for: Impact of Mycotoxins Secreted by Aspergillus Molds on the Inflammatory Response of Human Corneal Epithelial Cells
Source: Toxins (Basel). 2017 Jun 22;9(7):197. doi: 10.3390/toxins9070197 (PMC5535144; doi:10.3390/toxins9070197)
Supplement: Supplementary file 1 [file toxins-09-00197-s001.pdf]

Toxins **2016**, *21*, 197; doi:10.3390/toxins9070197

Supplementary Materials: Impact of Mycotoxins Secreted by *Aspergillus* Molds on the Inflammatory Response of Human Corneal Epithelial Cells

Yélian Marc Bossou, Youssra Serssar, Amel Allou, Sandrine Vitry, Isabelle Momas, Nathalie Seta, Jean Menotti and Sophie Achard

A

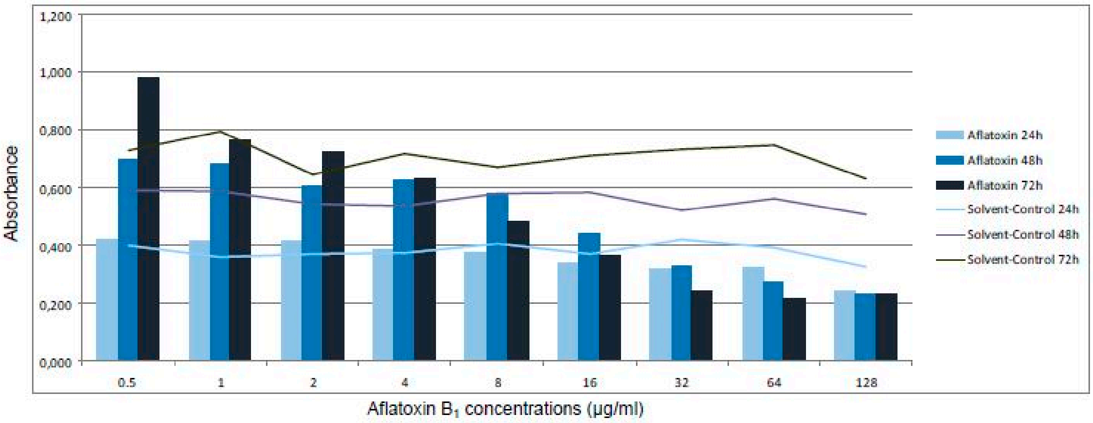

B

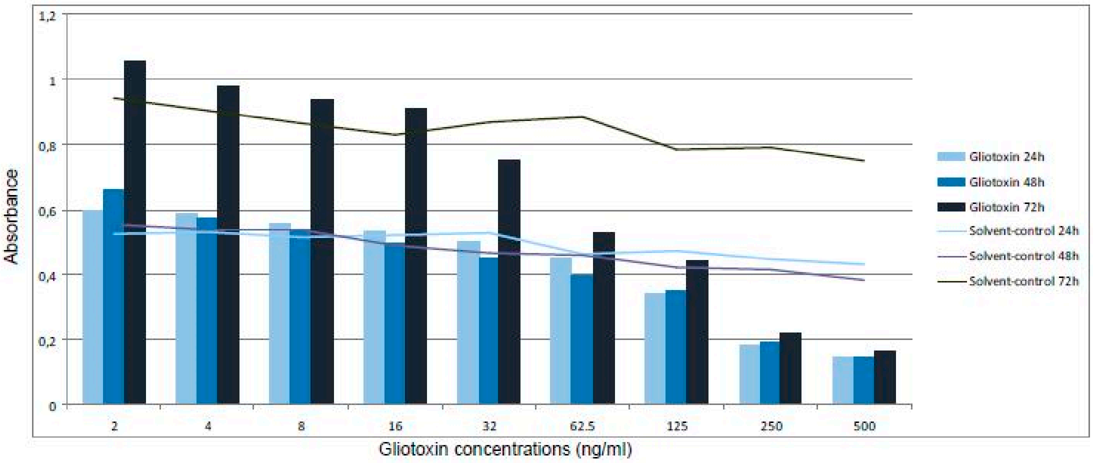

Figure S1. Cellular viability after 24 h, 48 h or 72 h of exposure to aflatoxin B_1, to gliotoxin or to DMSO alone using MTT assay. A. Cellular viability after exposure to nine different concentrations of aflatoxin B_1 (histograms) or to the nine concentrations of DMSO equivalent to the concentrations in the corresponding mycotoxin serial dilutions (curves). B. Cellular viability after exposure to nine different concentrations of gliotoxin (histograms) or to the nine concentrations of DMSO equivalent to the concentrations in the corresponding mycotoxin serial dilutions (curves). After 3 h of incubation with MTT at 37 °C and solubilization of formazan salts with DMSO, the absorbance was measured at 490 nm.

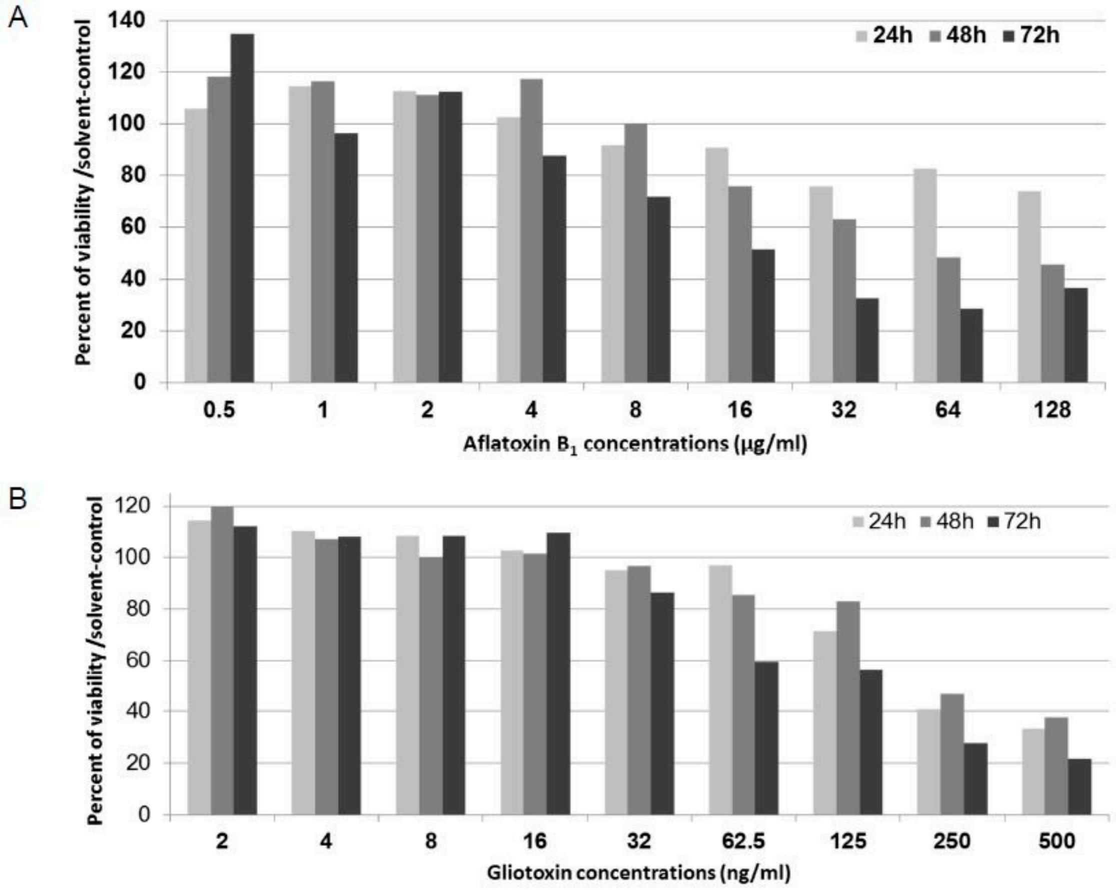

Figure S2. Cellular viability of HCE cells using MTT assay after 24 h, 48 h or 72 h of exposure to nine different concentrations of aflatoxin B_1 (**A**) or of gliotoxin (**B**). Results are expressed as percentage of viability compared to the corresponding concentration of DMSO (solvent-control).
